# Defining the region of interest of the knee for perioperative volumetric assessment with a portable 3D scanner in orthopedic and trauma surgery

**DOI:** 10.1371/journal.pone.0270371

**Published:** 2022-06-23

**Authors:** David Latz, Lisa Oezel, Roman Taday, Sebastian Viktor Gehrmann, Joachim Windolf, Erik Schiffner

**Affiliations:** Department of Orthopedics and Trauma surgery, Heinrich-Heine-University Duesseldorf, Duesseldorf, Germany; University Hospital Zurich, SWITZERLAND

## Abstract

**Background:**

The aim of this study was to characterize three regions of interest (ROI) around the knee with a portable 3D scanner (Artec 3D scanner EVA). Soft tissue topography assessment with an optimized, precise, and reproducible method may assist surgeons when managing soft tissue swelling in the post traumatic setting.

**Methods:**

12 healthy volunteers (24 legs, 7 women, 5 man) were included in this study. The patient cohort showed a mean age of 27.1 years (SD±3), a mean weight of 70 kg (SD±13) and a mean height of 171 cm (SD±8.8). All scans were recorded by the same examiner in the same room and with the same scanner (Artec, 3 D scanner EVA). Three volume regions of interest (ROI) were defined: the distal femur (circumference measured between the of superior extent of the patella to 10 cm proximal), the knee joint (measured from the top of the patella to the tibial tuberosity) and the proximal tibia (tibial tuberosity to 10 cm distal).

**Results:**

The mean volume of the right leg was 3.901 l (I. distal femur: 1.63 l, knee joint: 1.33 l, proximal tibia: 1.10 l) and mean volume of the left leg was 3.910 l (I. distal femur: 1.66 l, knee joint: 1.34 l, proximal tibia: 1.12 l). The volume difference between the right and left leg was 0.094 l (SD ± 0.083 l) The *Wilcoxon-Mann-Whitney test showed no significant differences* of the volumes between the right and left leg.

**Conclusions:**

This study demonstrates that portable 3D scanning could be an accurate and reliable tool for orthopedics and trauma surgeons. Based on the ROIs of this pilot study, further studies are needed to test the significance for clinical applications for patients with an injured knee.

## Introduction

Soft tissue swelling and edema are frequently encountered when managing orthopedic pathology [1]. The knee is prone to swelling secondary to tendinitis, arthritis, as well as any type of trauma such as fractures or ligament injuries. Severe swelling secondary to proximal tibial or distal femoral fractures precludes immediate operative intervention with open reduction and internal fixation and wrong timing of surgery being highly correlated with substantial soft tissue complications [2]. Therefore, a staged soft tissue management algorithm that begins with closed reduction and external fixation that is followed by open reduction and internal fixation is mandatory [2]. Tape and water displacement methods represent valid tools in the assessment of soft tissue swelling and yet are difficult to standardize and are subject to operator variability [3]. A gold standard for perioperative swelling characterization does not exist, which introduces a substantial subjective component regarding the timing of management for high energy injuries [2]. A portable three-dimensional (3D) scanner has been developed that measures the volume of a region of interest (ROI) [3, 4]. Previous studies have demonstrated that 3D scanning can efficiently achieve objective and reproducible measurements that correlate well with previously established tape measurement and water displacement methods [4]. The capabilities as they relate to perioperative traumatic soft tissue management have not been evaluated. The aim of this study was to employ a portable 3D scanner (Artec 3D scanner EVA) to determine three commonly encountered regions of interest (ROI) around the knee.

## Material and methods

### Population

12 healthy volunteers (24 legs, 7 women, 5 men) were included in this study. The patient cohort showed a mean age of 27.1 years (SD±3), a mean weight of 70 kg (SD±13) and a mean height of 171 cm (SD±8.8). Participants who documented injuries or any other functional disorders regarding knee, or ankle were excluded from the study. Each subject completed a standardized questionnaire (age, height, weight, gender, supporting vs. free leg) and an informed consent was obtained prior to the procedure. The study was performed according to the guidelines provided by the Declaration of Helsinki and was approved by the university ethical committee (STUDY NUMBER 2019–475).

### Image processing and 3D analysis

Artec EVA (Arctec Group, Luxemburg) uses a structured light triangulation methodology to noninvasively characterize the topography of the skin. During the scanning process, objects are illuminated and then scanned to recognize and record the region of interest (ROI) with two separate cameras. A third camera receives texture information. In order to process 3D scans, certain patterns that subdivide the ROI must be marked. Arctec EVA processes as many as 16 3D pictures per second, which are processed in real time using custom software (Artec Studio, Arctec Group, Luxemburg). A preview of the scanned object is immediately available. After the scanning process is complete, the picture and texture information is fused and merged by the software to create a color texturized 3D scan. The scan is received in a STL file and is exported to a computer as a Joint Photographic Experts Group File Interchange Format (.jpg) together with texture mapping information inside a Material Template Library file (.mtl).

### Study protocol and scanning procedure

**Fig 1:**
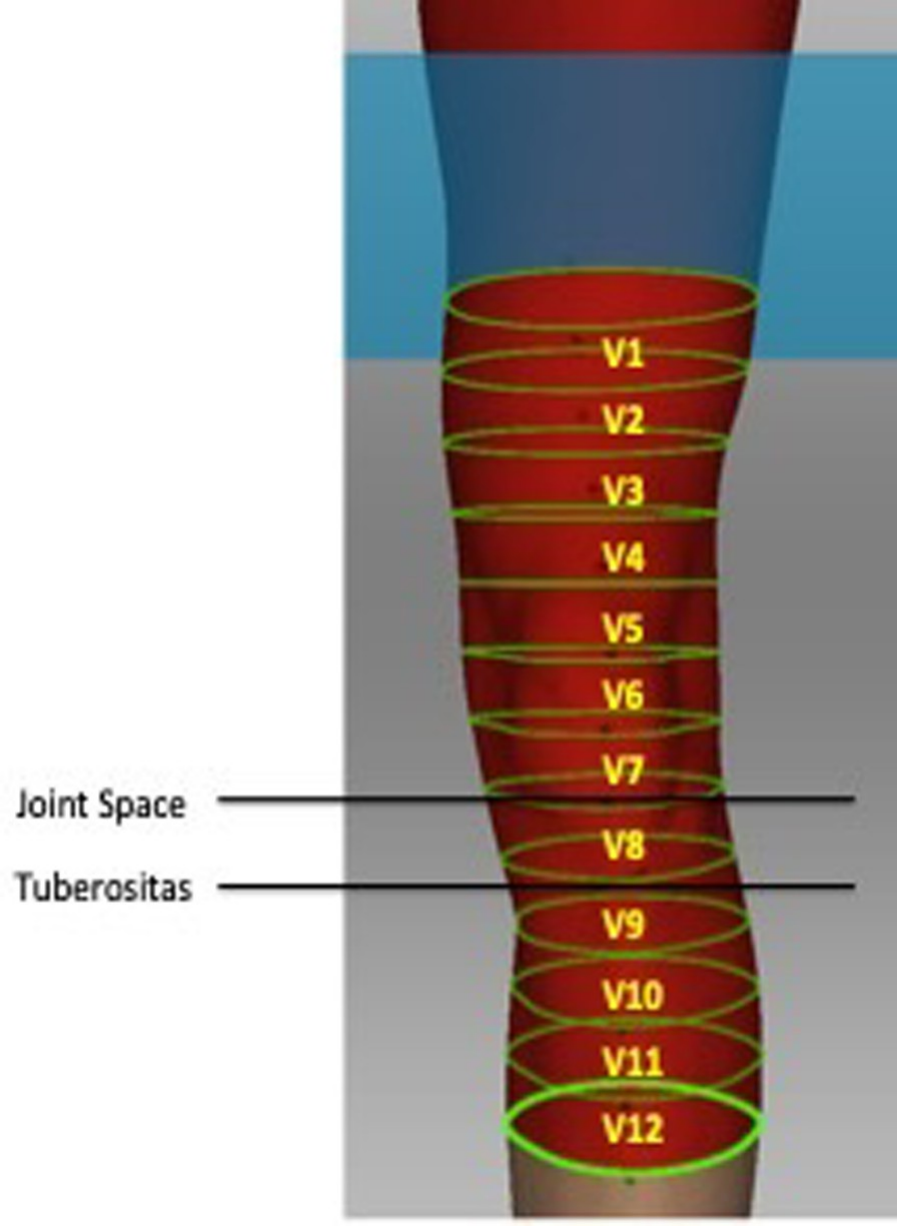
Volumes. 3D scanning of the knee indicating the measured volumes 20 cm proximal and 10 cm distal from the tibial tuberosity (starting point). The volumes were subdivided into segments of 2.5 cm.

All scans were recorded by the same examiner and took place in the same room and with the same scanner (Artec, Modell EVA). Before scanning, circumferences were indicated with a marker. Hereby, the tibial tuberosity was established as a beginning point and the circumferences were marked 20 cm proximal and 10 cm distal, subdivided in segments of 2.5 cm (12 Volumes-V, Fig 1).

**Fig 2:**
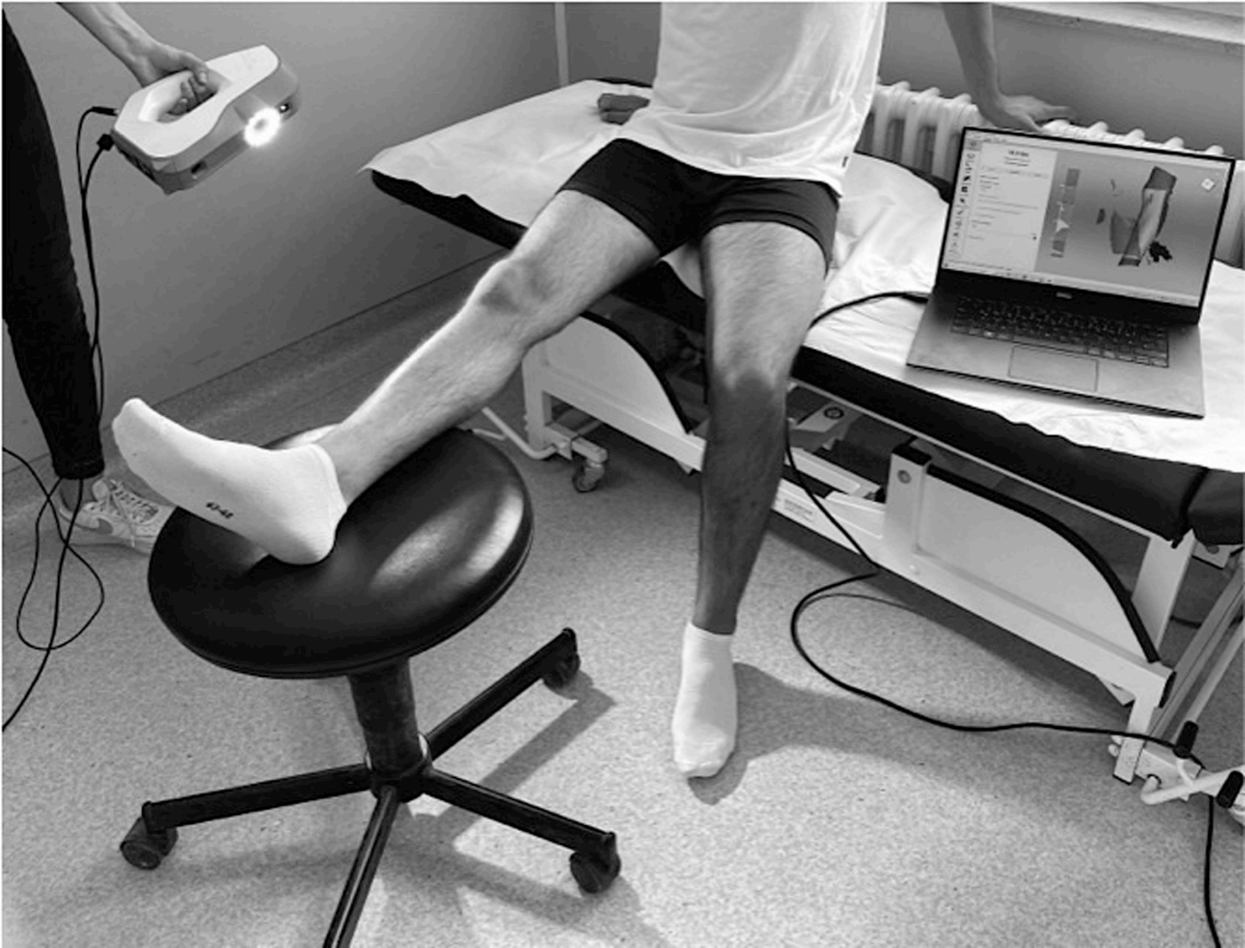
Scanner and workflow (own picture).

Volunteers were seated and their full extended legs were placed on a rest table. Volunteers were asked to keep a natural foot position (90 degree angle). After that, the scanning procedure was started, and the examiner moved the scanner around the volunteer (Fig 2) until the knee as well as the surrounding ROI were completely recorded. All volunteers were instructed not to move during the scans. The ideal distance to perform the best scan was determined by the distance adjustment indicator within the Artec Studio 13 Software (Version 13, Artec Group, Luxembourg).

**Fig 3:**
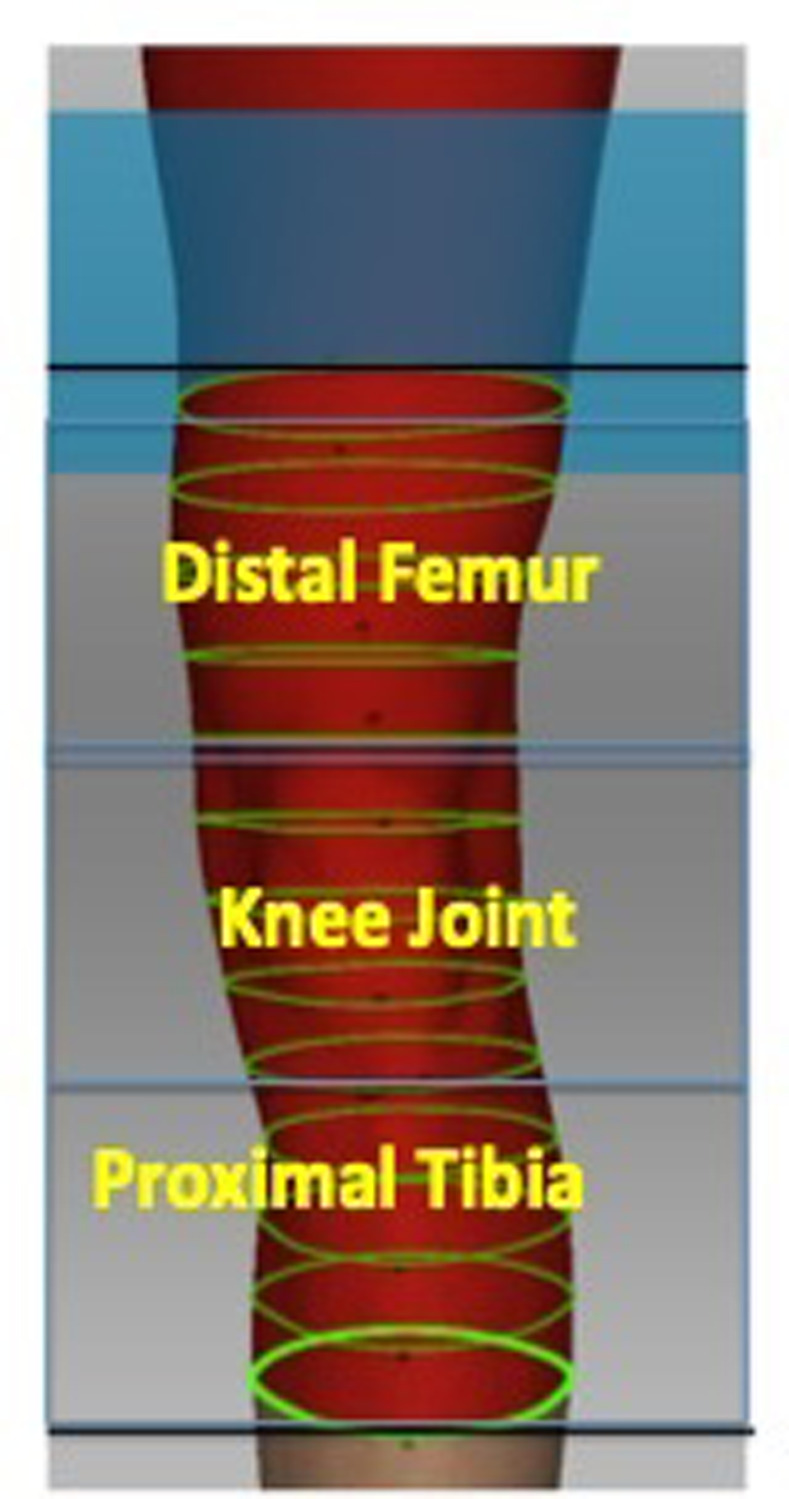
Region of interest. Determination of the three volume regions of interest (ROI). Distal Femur: from the top of the patella to 10 cm proximal from that (Volume 1–4), Knee Joint: from the top of the patella to the tuberositas tibiae (Volume 5–8), Proximal Tibia: from the tuberositas tibiae to 10 cm distal from that (Volume 9–12).

Each scanning took around 5.8 (SD ± 2) minutes. Three volume regions of interest (ROI, Fig 3) were defined. To evaluate the interobserver-reliability, all ROIs were determined by four different orthopaedic surgeons and compared to each other.

The distal femur was defined from the circumference of top of the patella to 10 cm proximal (Volume 1- Volume 4). The knee joint was defined from the top of the patella to tuberositas tibiae (Volume 5—Volume 8). The proximal tibia starts from tuberositas tibiae and ends 10 cm distal (Volume 9 –Volume 12). The mean distance from the tuberositas tibiae to the top of the patella in full extended legs in 12 cases was 11.4 cm (SD ± 1.8). The suprapatellar bursa is located proximal to the knee joint, between the prefemoral and suprapatellar fat pads. In most (~85%) people, the suprapatellar bursa communicates with the knee joint. The suprapatellar bursa does not communicate with the knee joint in ~15% of people, remaining separated by an embryonic septum [5, 6]. However, we included the suprapatellar bursa in the distal femur.

### Statistical analysis

Statistical analysis was performed using GraphPad Prism (Version 8.1.2, San Diego, California, USA). Data was first tested for normality using D’Agostino-Pearson normality test. Data which showed no normal distribution were further tested using Wilcoxon-Mann-Whitney signed-rank test to compare the volume differences between the left and right leg. P-values ≤ 0.05 were considered significant. An a priori power analysis was performed (G*Power Version 3.0.10, Franz Faul, University of Kiel, Germany). This resulted in a sample size of 10 for a power of 80% with a p value of 0.05 determining significance.

## Results

The collective consisted of 7 women and 5 men (mean age 27.1 years, range 23 to 34 years). Leg volumes (volume 1—volume 12) ranged from 2.77 liter to 6.11 liter. Each 3D measurement required around 5.8 ± 2 minutes. The results of reproducibility of these three ROIs by four raters were highly reliable (intraclass correlation coefficient (ICC) = 0.90, 95% confidence interval: 0.87–0.93)

### Comparison left vs right leg

**Fig 4:**
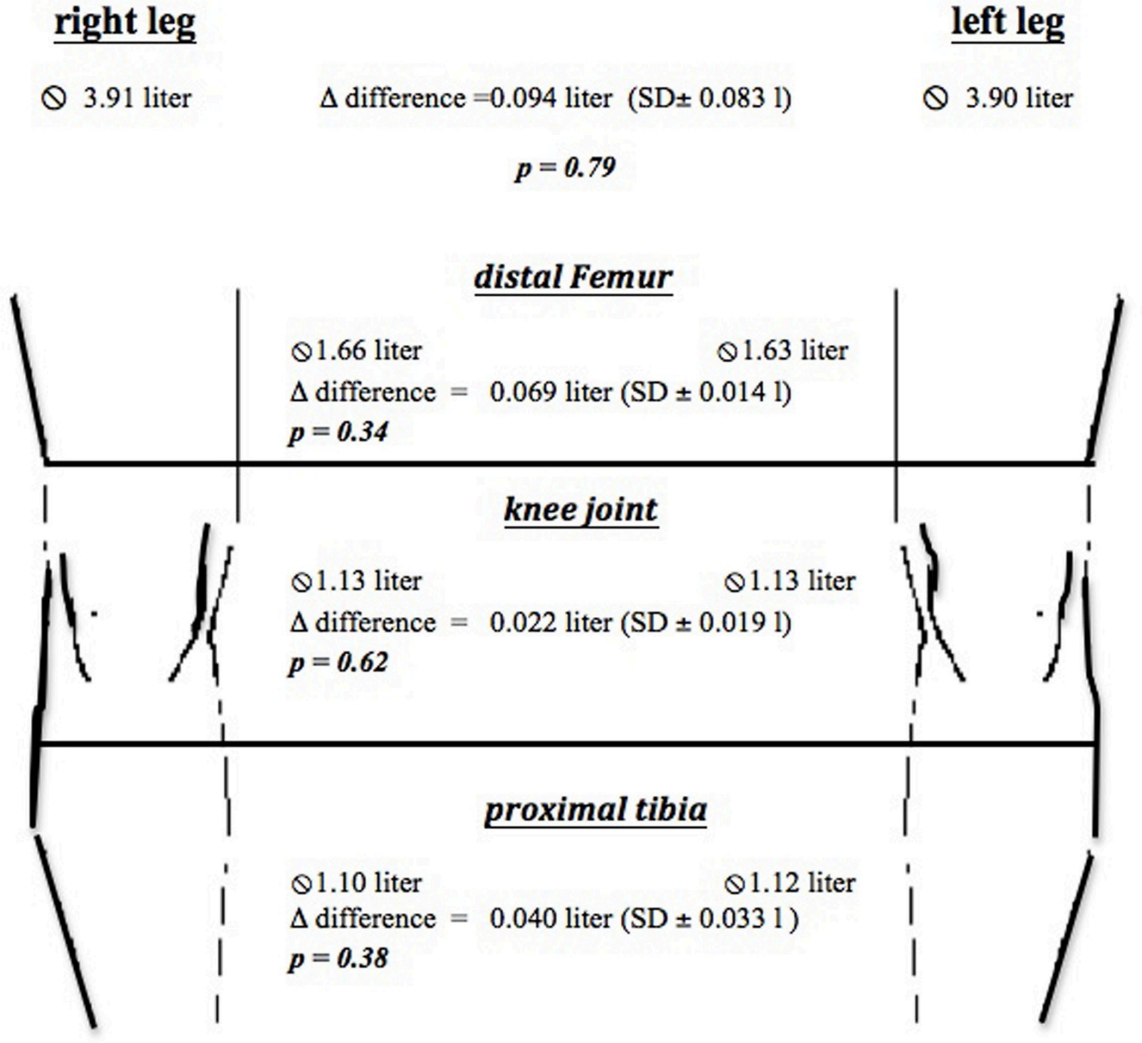
Comparison of the three ROI between right and left knee joint. 3D scanning of the knee indicating the measured volumes 20 cm proximal and 10 cm distal from the tibial tuberosity (starting point). The volumes were subdivided into segments of 2.5 cm.

Overall leg volume and volumes of the ROIs (I. distal femur, II. knee joint, III. proximal tibia) were compared between right and left leg (Fig 4).

The mean volume of the left leg was 3.90 ± 0.83 l (mean ± SD) (I. distal femur: 1.63 ± 0.38 l, knee joint: 1.13 ± 0.22 l, proximal tibia: 1.12 ± 0.23 l) and mean volume of the right leg was 3.91 ± 0.85 l (I. distal femur: 1.66 ± 0.42 l, knee joint: 1.13 ± 0.21 l, proximal tibia: 1.10 ± 0.21 l). The volume difference between the right and left leg was 0.094 l (SD ± 0.083 l) (I. distal femur: 0.069 l (SD± 0.051 l), II. knee joint: 0.022 l (SD ± 0.019 l), III. proximal tibia: 0.040 l (SD± 0.033 l)).

The Wilcoxon-Mann-Whitney test showed no significant differences of the volumes between the right and left leg (Overall leg volume: p = 0.79, I. distal femur: p = 0.34, II. knee joint: p = 0.62, III. proximal tibia: p = 0.38, Fig 3).

No significant volume differences between supporting and free leg were found (p>0.05).

## Discussion

Objective assessment of soft tissue and swellings is a substantial challenge to orthopedic and trauma surgeons. Incorrect characterization may affect the timing of surgery and is higly correlated with soft tissue complications. No reliable objective measurement method exists for determining peri operative swelling of the limbs. Although certain methods have been described including bioelectrical impedance, tape measurement and water displacement methods [3, 7–11], they are all difficult to perform on a trauma patient. In particular, water displacement and tape measurement albeit reliable [4, 12, 13] cannot be used with open wounds and is both time-consuming and cumbersome. Moreover, the water displacement method provides zero information about the shape of the injured extremity [4, 7, 14]. For all of the reasons, perioperative assessment of soft tissue swelling is still performed in a subjective manner and varies among surgeons. An ideal method for volume assessment of the limbs of injured patients should be valid, objective, reliable, non-invasive, fast and preferably without the use of radiation. Especially in trauma surgery, preoperative volume comparison to the contralateral healthy limb is crucial. It is believed that a novel 3D scanner may offer substantial advantages and compete with existing methods [3, 4].

Koban et al. tested the validity of the portable Artec Eva 3D scanner for medical purposes and showed a significant correlation to the water displacement method [3, 7]. Seminati et al. analyzed the mean percentage error of the Artec Eva scanner in comparison to the water displacement method and identified only a mean error of 1.4% [15].

However, there is a lack of information concerning the volume variability between contralateral limbs in healthy subjects. Therefore, evidence -based assessment of the volume of an injured knee in comparison to the contralateral healthy side is not possible. In contrast to the water displacement method, portable 3D scanners are capable to analyze a specific region of interest (ROI) and detect differences to the unharmed contralateral limb in this ROI. This is particularly helpful for staged soft tissue management in severely injured patient (e.g.distal femur fractures, proximal tibia fractures or intraarticular knee injuries). To assess soft tissues for typical injuries in orthopedics and trauma surgery, it was mandatory to define three typical ROI around the knee (I. distal femur, II. knee joint, III. proximal tibia). In Accordance to previous studies, landmarks for the ROI with the highest inter- and intraobserver reliability were chosen [16]. Our results showed no significant differences of the overall volume and all three ROI between right and left leg. Accordingly, the selected ROIs seem to be valid for assessing and comparing injured limbs to the healthy contralateral side. In the present study, scanning time for both knees was approximately 5.8 (SD± 2) minutes and not significantly longer than for other medical purposes like assessment of lymphedema in previous studies [4]. Therefore, our data are in line with previous studies and suggest, that soft tissue assessment around the knee with the portable Artec Eva 3D scanner could be faster than conventional tape measurement and water displacement measurement [4]

This study has several limitations. One limitation of this study is the small sample size of 12 healthy participants. The portable Arctec 3D scanner has a resolution of 0.1mm and previous studies with a similar sample size have shown a mean percentage error of 1.4% when compared to other methods [15]. Another limitation is that only one specific camera system was used for volumetric assessment. The Artec Eva 3D scanner has been previously used for medical purposes and showed a significant correlation to the goldstandard volumetric assessment [3, 7, 15]. A limitation of this technology is that scanning of a patient in a splint is not possible. Patients with an external fixation can be scanned as Artec software offers the ability to to substract external fixation after the scanning process has been completed. Finally, only leg volume of healthy volunteers was measured. Aim of this pilot study was to determine ROI of the knee that can be used for the most types of injuries. Therefore, our results are going to use for future studies to evaluate injured patients and therapeutic strategies over time.

## Conclusion

This study demonstrates that portable 3D scanning may be an accurate and reliable tool for orthopedics trauma surgeons. Improved soft tissue management may improve the outcomes of severely injured knees. Subsequent studies that include injured patients are required to validate the ROIs.

## References

[1] Schellong SM, Wollina U, Unger L, Machetanz J, Stelzner C. [Leg swelling]. Der Internist. 2013;54(11):1294–303. Epub 2013/11/23. doi: 10.1007/s00108-013-3339-z . 24264570

[2] Canton G, Santolini F, Stella M, Moretti A, Surace MF, Murena L. Strategies to minimize soft tissues and septic complications in staged management of high-energy proximal tibia fractures. European journal of orthopaedic surgery & traumatology: orthopedie traumatologie. 2020. Epub 2020/01/02. doi: 10.1007/s00590-019-02619-9 . 31893294

[3] Koban KC, Titze V, Etzel L, Frank K, Schenck T, Giunta R. [Quantitative volumetric analysis of the lower extremity: validation against established tape measurement and water displacement]. Handchirurgie, Mikrochirurgie, plastische Chirurgie: Organ der Deutschsprachigen Arbeitsgemeinschaft fur Handchirurgie: Organ der Deutschsprachigen Arbeitsgemeinschaft fur Mikrochirurgie der Peripheren Nerven und Gefasse 2018;50(6):393–9. Epub 2019/01/09. doi: 10.1055/a-0770-3445 . 30620977

[4] Landau MJ, Kim JS, Gould DJ, Patel KM. Vectra 3D Imaging for Quantitative Volumetric Analysis of the Upper Limb: A Feasibility Study for Tracking Outcomes of Lymphedema Treatment. Plastic and reconstructive surgery. 2018;141(1):80e–4e. Epub 2017/09/19. doi: 10.1097/PRS.0000000000003912 . 28922322

[5] Steinbach LS, Stevens KJ. Imaging of cysts and bursae about the knee. Radiologic clinics of North America. 2013;51(3):433–54. Epub 2013/04/30. doi: 10.1016/j.rcl.2012.10.005 . 23622093

[6] Zidorn T, Schäfer H. Morphologic variants of the proximal knee-joint cavity. An anatomical and radiological study. Surgical and radiologic anatomy: SRA. 1992;14(2):141–6. Epub 1992/01/01. doi: 10.1007/BF01794891 . 1641739

[7] Hameeteman M, Verhulst AC, Vreeken RD, Maal TJ, Ulrich DJ. 3D stereophotogrammetry in upper-extremity lymphedema: An accurate diagnostic method. Journal of plastic, reconstructive & aesthetic surgery: JPRAS. 2016;69(2):241–7. Epub 2015/11/23. doi: 10.1016/j.bjps.2015.10.011 . 26590631

[8] Hidding JT, Viehoff PB, Beurskens CH, van Laarhoven HW, Nijhuis-van der Sanden MW, van der Wees PJ. Measurement Properties of Instruments for Measuring of Lymphedema: Systematic Review. Physical therapy. 2016;96(12):1965–81. Epub 2016/06/25. doi: 10.2522/ptj.20150412 . 27340195

[9] Knoops PG, Beaumont CA, Borghi A, Rodriguez-Florez N, Breakey RW, Rodgers W, et al. Comparison of three-dimensional scanner systems for craniomaxillofacial imaging. Journal of plastic, reconstructive & aesthetic surgery: JPRAS. 2017;70(4):441–9. Epub 2017/02/06. doi: 10.1016/j.bjps.2016.12.015 . 28161205

[10] Spanholtz TA, Leitsch S, Holzbach T, Volkmer E, Engelhardt T, Giunta RE. [3-dimensional imaging systems: first experience in planning and documentation of plastic surgery procedures]. Handchirurgie, Mikrochirurgie, plastische Chirurgie: Organ der Deutschsprachigen Arbeitsgemeinschaft fur Handchirurgie: Organ der Deutschsprachigen Arbeitsgemeinschaft fur Mikrochirurgie der Peripheren Nerven und Gefasse 2012;44(4):234–9. Epub 2012/08/31. doi: 10.1055/s-0032-1316379 . 22932855

[11] Tierney S, Aslam M, Rennie K, Grace P. Infrared optoelectronic volumetry, the ideal way to measure limb volume. European journal of vascular and endovascular surgery: the official journal of the European Society for Vascular Surgery. 1996;12(4):412–7. Epub 1996/11/01. doi: 10.1016/s1078-5884(96)80005-0 . 8980428

[12] Karges JR, Mark BE, Stikeleather SJ, Worrell TW. Concurrent validity of upper-extremity volume estimates: comparison of calculated volume derived from girth measurements and water displacement volume. Physical therapy. 2003;83(2):134–45. Epub 2003/02/05. . 12564949

[13] Boland R, Adams R. Development and evaluation of a precision forearm and hand volumeter and measuring cylinder. Journal of hand therapy: official journal of the American Society of Hand Therapists. 1996;9(4):349–58. Epub 1996/10/01. doi: 10.1016/s0894-1130(96)80041-x . 8994010

[14] Sander AP, Hajer NM, Hemenway K, Miller AC. Upper-extremity volume measurements in women with lymphedema: a comparison of measurements obtained via water displacement with geometrically determined volume. Physical therapy. 2002;82(12):1201–12. Epub 2002/11/26. . 12444879

[15] Seminati E, Canepa Talamas D, Young M, Twiste M, Dhokia V, Bilzon JLJ. Validity and reliability of a novel 3D scanner for assessment of the shape and volume of amputees’ residual limb models. PloS one. 2017;12(9):e0184498. Epub 2017/09/09. doi: 10.1371/journal.pone.0184498 ; PubMed Central PMCID: PMC5590959. 28886154 PMC5590959

[16] Tunc R, Caglayan-Tunc A, Kisakol G, Unler GK, Hidayetoglu T, Yazici H. Intraobserver and interobserver agreements of leg circumference measurements by tape measure based on 3 reference points. Angiology. 2007;58(5):593–6. Epub 2007/11/21. doi: 10.1177/0003319707307844 . 18024943

